# Genus-Specific Real-Time PCR and HRM Assays to Distinguish *Liriope* from *Ophiopogon* Samples

**DOI:** 10.3390/plants6040053

**Published:** 2017-10-26

**Authors:** Eva Masiero, Dipanwita Banik, John Abson, Paul Greene, Adrian Slater, Tiziana Sgamma

**Affiliations:** 1 Biomolecular Technology Group, Allied Health Sciences, Faculty of Health and Life Sciences, De Montfort University, Leicester LE1 9BH, UK; eva.masiero@dmu.ac.uk (E.M.); banikdipanwita@yahoo.com (D.B.); ads@dmu.ac.uk (A.S.); 2 CSIR-North East Institute of Science & Technology, Jorhat 785006, Assam, India; 3 Environmental Industries and Business, Brooksby Melton College, Brooksby Campus, Brooksby, Leicestershire LE14 2LJ, UK; johnabson42@gmail.com (J.A.); pgreene@BrooksbyMelton.ac.uk (P.G.)

**Keywords:** *Ophiopogon*, *Liriope*, rbcL, DNA barcoding, high-resolution melt curve (HRM) analysis

## Abstract

*Liriope* and *Ophiopogon* species have a long history of use as traditional medicines across East Asia. They have also become widely used around the world for ornamental and landscaping purposes. The morphological similarities between *Liriope* and *Ophiopogon* taxa have made the taxonomy of the two genera problematic and caused confusion about the identification of individual specimens. Molecular approaches could be a useful tool for the discrimination of these two genera in combination with traditional methods. Seventy-five *Liriope* and *Ophiopogon* samples from the UK National Plant Collections of *Ophiopogon* and *Liriope* were analyzed. The 5′ end of the DNA barcode region of the gene for the large subunit of ribulose-1,5-bisphosphate carboxylase/oxygenase (*rbcLa*) was used for the discrimination of the two genera. A single nucleotide polymorphism (SNP) between the two genera allowed the development of discriminatory tests for genus-level identification based on specific PCR and high-resolution melt curve (HRM) assays. The study highlights the advantage of incorporating DNA barcoding methods into plant identification protocols and provides simple assays that could be used for the quality assurance of commercially traded plants and herbal drugs.

## 1. Introduction

Plants belonging to the genera *Liriope* Lour. and *Ophiopogon* Ker Gawl. are collectively known by the English common name liriopogon [1,2,3]. The collective name itself indicates the close relationship and morphological similarities between the two genera and the potential for misidentification. Liriopogon are widely cultivated as ornamentals and ground cover plants for garden landscaping due to their hardiness, and pest and disease resistance. However, mishandling, mislabelling, and mismanagement of nursery practices can lead to sexual propagation of cultivars, hybridisation, plant substitution, and finally degradation of the morphological/phenotypic identity of the cultivars [4].

Tubers of a few species of both *Liriope* and *Ophiopogon* are used in traditional medicines across East and South Asia for the treatment of respiratory ailments, diabetes, thirst, and as an aphrodisiac [5]. In the Chinese and Korean traditions, substitution of *Liriope* for *Ophiopogon* is permissible, although the Chinese Pharmacopoeia considers them to be separate remedies [6]. In contrast, the Japanese Pharmacopoeia stipulates that the traditional medicine “Bakumondo” must be derived from *O. japonicus* tubers, i.e., material derived from *Liriope* is not a legal substitute. The close similarity in the morphological characteristics of their leaves and tubers makes it difficult to distinguish between members of the two genera in both the horticultural and phytopharmaceutical industries [4]. Methods for discrimination of samples from the two genera are therefore important for quality assurance in these commercial sectors.

Authentication of plant material used for herbal medicines typically relies on chemical analysis. Liriopogons are characterised by their content of steroidal saponins and homoisoflavonoids [6,7,8]. TLC methods are straightforward and suitable for multiple samples. A TLC assay to distinguish the two genera has been developed, but is limited by low sensitivity and resolution [9]. More precise analysis of the saponin and flavonoid components has been achieved by HPLC-UV [10] and HPLC-UV-ELSD [8], but these require a long run time for each individual sample. Recent comparison of the two genera by LC-MS/MS also showed that differences in the profiles of steroidal saponins and homoisoflavonoids could be used to discriminate between *Ophiopogon* and *Liriope* [6].

DNA-based tests have emerged as a powerful system for authentication of medical plants and commercial herbal products [11,12,13]. Many of these target “DNA barcode” regions of the plant genome. DNA barcoding is a technique for identifying biological specimens using short DNA sequences from either the nuclear or organelle genome, termed DNA barcodes. In plants, the major DNA barcode candidates are the plastid *matK*, *rbcL*, and *trnH-psbA* loci and the nuclear ribosomal ITS region (nrITS) [14,15,16,17]. DNA tests have been developed to distinguish *Liriope* from *Ophiopogon*, including the use of SCAR [18] and EST-SSR [19] markers. A barcoding approach targeting a SNP in the 3′ region of the rbcL region was developed by Ito et al. (2015) [20]. Digestion with the restriction enzyme *Hinc*III cut amplicons from *Liriope* into two fragments, but left *Ophiopogon* amplicons intact. This is an effective assay, but the digestion and gel electrophoresis steps are time-consuming and limit the throughput of the assay.

The current study proposes a new strategy for the identification of *Ophiopogon* and *Liriope* species by designing specific real-time PCR and high-resolution melt curve (HRM) assays targeting genus-specific single nucleotide polymorphisms (SNPs) in the *rbcL* barcode region. These techniques are ideally suited for the design of quick, reliable, robust, and affordable identification assays that could be incorporated into industrial quality control procedures for herbal medicines [13].

This project arose from a collaboration to verify the identity of accessions in the UK National Collections of *Ophiopogon* and *Liriope* by DNA barcoding [21]. However, to date, there are few examples of DNA techniques being applied to the classification of cultivated ornamental plants. An identification test based on DNA barcoding would be much faster than the traditional botanical methods of identification, which require growing the target plants to the flowering stage, in parallel with control plants. This new affordable method could also be useful for nurseries and plant collections and the wider horticultural community of professional and amateur gardeners.

## 2. Results

### 2.1. DNA Barcoding of the rbcLa Region of Liriope and Ophiopogon Accessions

**Figure 1:**
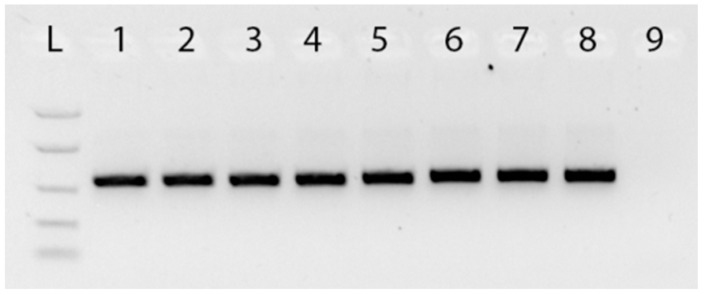
Agarose gel electrophoresis of PCRs using *rbcL* generic primers. Gel lanes: L. Easy Ladder I (Bioline) 1. *Ophiopogon* Sample 678; 2. *Ophiopogon* Sample 679; 3. *Ophiopogon* Sample 680; 4. *Ophiopogon* Sample 682; 5. *Liriope* Sample 626; 6. *Liriope* Sample 627; 7. *Liriope* Sample 631; 8. *Liriope* Sample 632; 9. Negative (no template) control.

The plastid *rbcLa* barcode region of 75 National Collection *Ophiopogon* and *Liriope* specimens was analysed (Table S1). Genomic DNA was extracted from all the samples and amplified by conventional PCR using *rbcLa* generic primers. The expected fragment of about 700 bp was clearly visualised in all of the *Ophiopogon* and *Liriope* samples (Figure 1).

**Figure 2:**
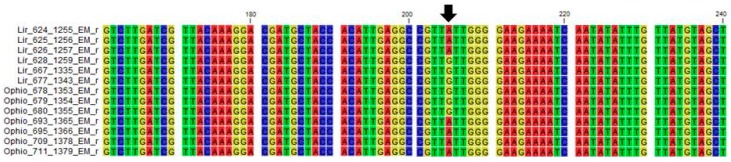
Fragment of a multiple alignment of the rbcL region from a selection of *Liriope* and *Ophiopogon* samples, highlighting a number of atypical samples. *Liriope* Samples 624, 625, and 626 match the consensus *Liriope* sequence. *Ophiopogon* Samples 678, 679, and 680 match the *Ophiopogon* consensus. *Liriope* Samples 628, 667, and 677 and *Ophiopogon* Samples 693, 695, 709, and 711 are atypical in the SNP position highlighted by the black arrow.

In order to confirm the genus and the species, the *rbcLa* amplicons were sequenced from the *rbcLa* primer. A multiple sequence alignment was generated, combining sequences from the GenBank database with the newly generated sequences (Figure S1). The alignment showed very little sequence variation between species of the same genus, with just three single nucleotide polymorphic (SNP) positions observed. One was found to vary between the two genera, with the guanine predominantly present in the *Ophiopogon* samples substituted by an adenine in the *Liriope* samples (Figure 2).

Only four *Ophiopogon* samples (693, 695, 709, and 711) and three *Liriope* samples (628, 667, and 677) did not fit this SNP pattern, having instead an adenosine and a guanine, respectively (Figure 2). One explanation is that these accessions had been misidentified. Three of the atypical *Ophiopogon* accessions had been classified as the same species, *O. japonicus,* but the three atypical *Liriope* accessions were originally classified as different species: *L. graminifolia*, *L muscari*, and *L spicata*. Morphological analysis of these specimens was not able to resolve this, since the vegetative aerial parts share common morphological characters (Figure S2).

In order to resolve the anomaly, the identity of these specimens was determined by DNA barcoding of the nrITS region (data not shown). The nrITS sequences of all seven specimens confirmed the genus assignment indicated by the *rbcLa* SNP, i.e., the plants did appear to have been mislabelled or misidentified. The consistent genus-specificity of the SNP confirmed by these results presented an opportunity to discriminate the *Ophiopogon* and *Liriope* genera by designing specific PCR primers to target this SNP.

### 2.2. The rbcL Feature Provides a Target for Simple Genus Discriminatory Tests

**Figure 3:**
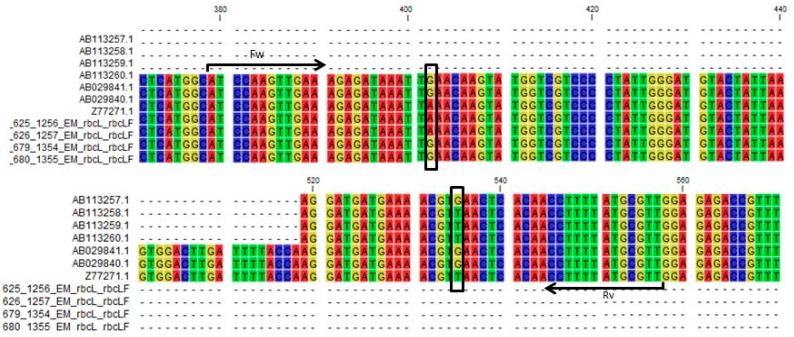
Graphical representation of the location of genus-specific primers. The figure shows a multiple alignment with 4 *Liriope* and 4 *Ophiopogon rbcLa* consensus sequences joined to the *rbcLb* sequences published by Ito et al. (2012) using three *rbcL* sequences from the database that bridge the *rbcLa* and *rbcLb* regions. The black arrow indicates where the forward and reverse primers were designed. The black boxes indicate the two SNPs incorporated into the 3′ position of the primer sequences.

In a study conducted by Ito et al. (2015) [21] it was reported that the two genera showed a single nucleotide variation in the *rbcLb* region and they designed a restriction-enzyme-based assay to target their SNP in order to discriminate the two genera. Their SNP is about 120 bp downstream from the one reported here. In order to develop a new and more robust assay for discriminating the two genera, two sets of genus-specific primers were designed to incorporate both SNPs. Thus, the *Ophiopogon*-specific forward primer was designed to end with the guanine base, while the reverse primer was designed to end with a cytosine corresponding to the guanine in the plus strand. Conversely, the *Liriope*-specific forward primer was designed to end with the adenosine base, while the reverse primer was designed to end with an adenosine corresponding to the thymidine in the plus strand (Figure 3).

**Figure 4:**
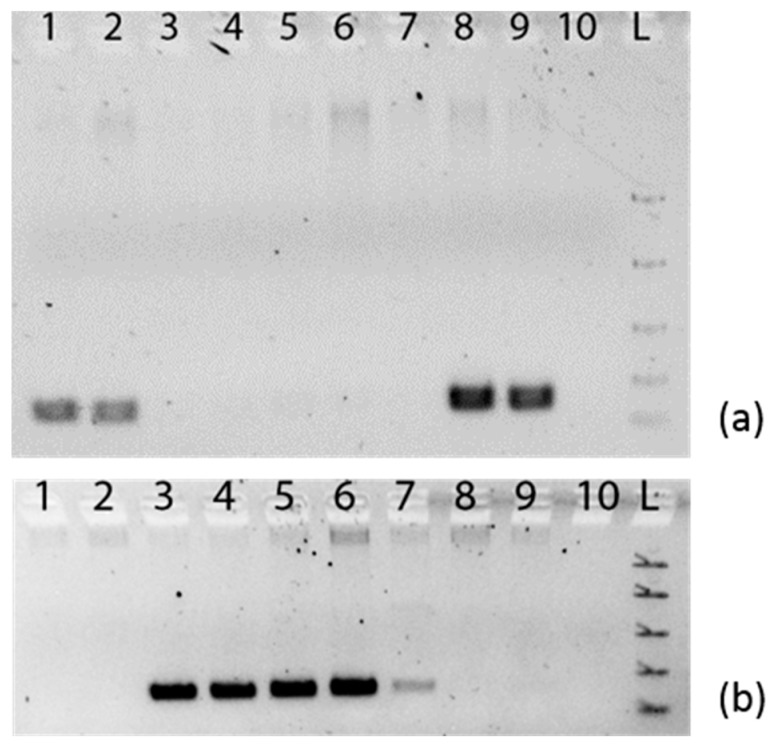
Agarose gel electrophoresis of PCR using rbcL_Ophiopogon and rbcL_Liriope specific primers. (**a**) rbcL_Ophiopogon specific primers. (**b**) rbcL_Liriope specific primers. Gel lines: 1. *Ophiopogon* Sample 678; 2. *Ophiopogon* Sample 679; 3. *Ophiopogon* Sample 693; 4. *Ophiopogon* Sample 695; 5. *Ophiopogon* Sample 709; 6. *Ophiopogon* Sample 711; 7. *Liriope* Sample 624; 8. *Liriope* Sample 628; 9. *Liriope* Sample 677; 10. Negative (no template) control; L. Easy Ladder I (Bioline).

Conventional PCR was performed with samples from the target and non-target genera in order to confirm the specificity of the primers (Figure 4). The annealing temperature of the PCR was optimised for each specific primer pair such that a prominent amplicon was produced with the correct template, but no product was visible with a template from the opposite genus. For example, in Figure 4a, DNA from the confirmed *Ophiopogon* Samples 678 and 679 (Lane 1 and 2) produce clear bands with the Ophiopogon-specific primers, but no bands are seen in these lanes in Figure 4b.

The assay was then used to test those samples that showed a different nucleotide base compared to their original classification. The *Ophiopogon*-specific primers clearly showed that Samples 693, 695, 709, and 711 did not belong to the genera to which they had been first allocated (Figure 4a), and the *Liriope*-specific primers confirmed that these samples belonged to the *Liriope* genus. In the same way, the specific assays confirmed that Samples 628 and 677 belong in the *Ophiopogon* genus and not in the *Liriope* genus to which they were originally assigned (Figure 4b).

To confirm the accuracy of the initial sampling of these specimens, a repeat collection of samples was carried out and the repeat DNA extractions were tested with the specific PCR assay. The results again confirmed that Samples 693, 695, and 709 belong to the *Liriope* genus and 628, 667, and 677 to *Ophiopogon* (Figure S3a,b). Our new discriminatory test, using genus-specific primers, permits us to identify *Ophiogon* and *Liriope* samples with an easy and economic system by conventional PCR.

**Figure 5:**
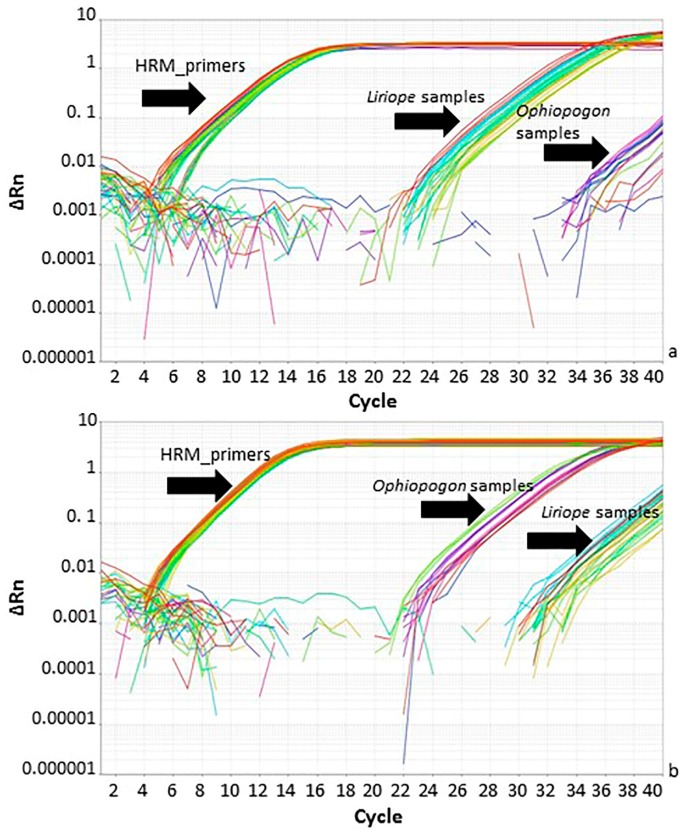
Real-time PCR amplification using *Ophiopogon*-specific, *Liriope*-specific and generic high-resolution melt curve (HRM) primers. (**a**) Amplification plot of *Liriope*-specific and HRM primers. (**b**) Amplification plot with *Ophiopogon*-specific and HRM primers. The black arrows indicate the primer/template combinations: HRM_primers: *Ophiopogon* and *Liriope* templates with the generic primers; *Liriope* samples: *Liriope* templates with specific primers; *Ophiopogon* samples: *Ophiopogon* templates with specific primers.

The specific primers targeted two genus-specific SNPs within a short region of the *rbcL* barcode sequence and were designed so that they were also suitable for use in real-time PCR analysis. The speed, simplicity, and sensitivity of real-time PCR assay are ideally suited to industrial quality control tests [13]. Real-time PCR was performed using the genus-specific primers after optimising the thermocycling programs and primer concentrations. The amplification plots for the *Liriope*-specific primers showed a marked difference in *C*_t_ value (around 12 cycles) between *Liriope* and *Ophiopogon* samples (Figure 5a). The *Ophiopogon*-specific primers were less efficient, but careful optimisation of the annealing temperature allowed a difference in *C*_t_ values between the two genera of around 10 cycles to be achieved. (Figure 5b).

In order to normalise the *C*_t_ values to allow for differences in the amount of DNA template, the HRM primers described in the next section were tested for their suitability as generic/universal primers. Real-time PCR with the HRM_primers did not show any significant variation in the *C*_t_ values obtained from samples from the two genera. The *C*_t_ value obtained with the generic primers was subtracted from the specific primer *C*_t_ to obtain a Δ*C*_t_ value for comparison between different DNA samples. In order to identify an unknown sample as *Liriope* or *Ophiopogon*, the Δ*C*_t_ value for a reference sample was subtracted from the unknown to produce a ΔΔ*C*_t_ value. It is expected that the ΔΔ*C*_t_ value from the correct specific primers will be ≤2.0, whilst that for an incorrect genus would be >7.0. A ΔΔ*C*_t_ > 7.0 is arbitrarily chosen as the threshold because it represents the equivalent of detection of the correct template at a dilution of one molecule in one thousand. The results in Table 1 clearly show that the ΔΔ*C*_t_ values correspond to the genus identity of each sample.

Thus, for example, each known or suspected *Liriope* sample produces a ΔΔ*C*_t_ value close to zero with the *Liriope* primers, whilst the *Ophipogon* primers produces a corresponding ΔΔ*C*_t_ value above seven.

### 2.3. Identification of Liriope and Ophiopogon Samples by Using the HRM System

**Figure 6:**
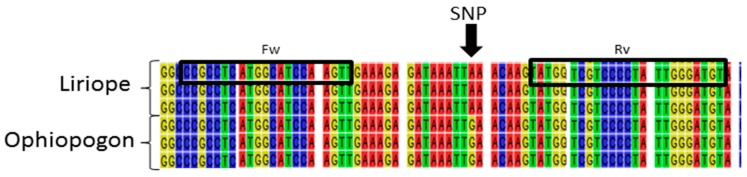
Schematic representation of the location of the HRM primers. The black arrow indicate the position of the SNP.

The *rbcLa* SNP also provides a useful target for developing a single tube assay to discriminate between the two genera using HRM analysis. This technology can discriminate between sequences containing a difference of a single base if it has a significant effect on the melting temperature. HRM primers were designed to the conserved regions of the *rbcL* sequence on either side of the SNP (Figure 6).

After the optimisation of the PCR conditions by conventional PCR (Figure S4), the HRM curves produced from *Liriope* and *Ophiopogon* samples were compared. The results revealed that there was a difference in Tm of 2 °C, allowing the two genera to be easily distinguished from each other. A difference plot of the melting curves showed two distinct variants that corresponded to samples from the two genera (Figure S5).

**Figure 7:**
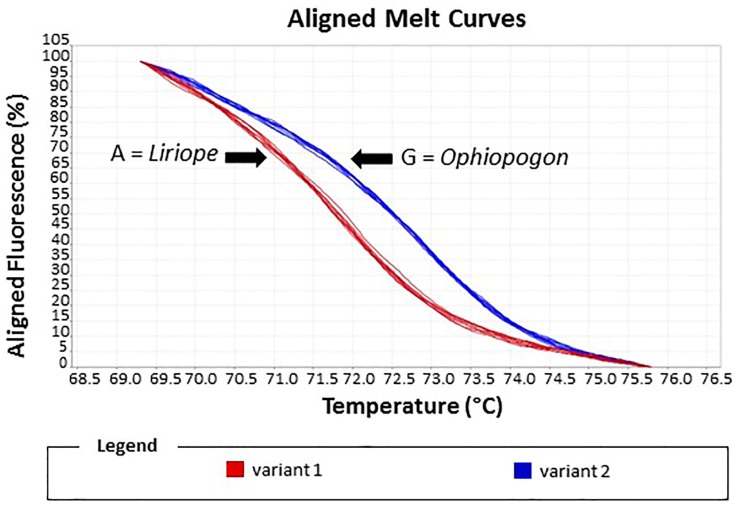
HRM assay of reference standards and test samples. The melting curve plot showed two distinct variants: Variant 1, *Liriope* standards; Variant 2, *Ophiopogon* standards. Test samples were assigned to the *Liriope* or *Ophiopogon* genus according to which variant curve they matched.

The reliability of the assay was assessed by a blind experiment in which the identities of ten samples were unknown to the operator. Alignment of the melting curves allowed the two variants to be easily discriminated. In Variant 1, four unknown samples were matched with the *Ophiopogon* controls, whilst 6 unknown samples in Variant 2 matched the *Liriope* controls (Figure 7). These ten samples included the seven misidentified samples.

This assay confirmed the correct genus of these samples, supporting the results of the genus-specific real-time PCR assay.

## 3. Discussion

Sequencing of the *rbcLa* barcode region of 75 samples of *Ophiopogon* and *Liriope* from the UK National Plant Collections of these two genera provided a large dataset for analysis. Multiple alignment of the sequences revealed that the region is very highly conserved, with only three SNPs observed, one of which distinguishes the genus *Liriope* from the genus *Ophipogon*. This reflects the findings of [22] who reported the high degree of conservation of the *rbcLb*, though they observed five genus-specific SNPS in this downstream section of the gene. The *rbcLb* region has been noted as being more variable than the *rbcLa* region in a number of plant groups [23]. Ito et al. (2015) [20] targeted one of these genus-specific *rbcLb* SNPs using a restriction-enzyme-digestion-based approach for the identification of the two genera. This SNP was around 100 bases downstream of ours in the *rbcLb* region, so proved ideal for the design of pairs of specific real-time PCR primers.

Our analysis of the sequence data indicated that our *rbcLa* SNP might not be entirely genus-specific because three *Liriope* and four *Ophiopogon* samples had the “wrong” base at this position. There are a number of possible biological explanations for this including homoplasy and hybridisation, but human errors of identification or labelling appeared more likely. Sequencing of the nrITS barcode region of these specimens showed that the genus identification agreed with the *rbcL* SNP, indicating that they had been misidentified (data not shown). This confirmed that the SNP was entirely genus-specific across the collection.

In order to develop rapid, reliable *Ophiopogon* and *Liriope* identification tests, the SNP identified in our study and that targeted by Ito et al. (2015) [20] allowed the design of genus-specific primers for a simple PCR-based test. The primers were designed to incorporate the variable base at the 3′ end of the primer for conventional and real-time PCR. This strategy has been used in the design of a number of PCR tests for the authentication of herbal medicines [13,15]. The specificity of the primers was confirmed by conventional PCR; bands were only seen with template DNA from the corresponding genus.

The genus-specific primers were also designed for use in real-time PCR. The *Liriope*-specific primers could distinguish DNA from the two genera by a difference in *C*_t_ value of 12 cycles, when normalised with the HRM generic primers. The *Ophiopogon* primers showed a similarly large difference in *C*_t_ values after the annealing temperature of the PCR was optimised for specificity. It is noticeable that the *C*_t_ values obtained with the specific primer pairs were considerably higher than the *C*_t_ obtained with the generic HRM primers. This is the result of a number of factors. The design of the specific primers is constrained by the position of the polymorphic base at the 3′ end. The only flexibility in design is variation of the total length of the primer. In addition, the optimisation of specificity often requires that the annealing temperature of the PCR is higher than optimal for amplification efficiency. Nevertheless, the results indicate that the design constraints and sub-optimal conditions do not affect the ability of the assay to identify *Ophiopogon* and *Liriope* samples in a quick and consistent way. The value of the assay was highlighted when it was used to rapidly confirm the genus of the misidentified accessions after resampling.

Under optimal conditions, HRM assays can discriminate between sequences containing a difference of a single base and can rapidly and accurately identify species from a diverse range and quality of materials [13]. In our study, the HRM assay also proved to be a simple and reliable method for the identification of the two genera. The results grouped the samples into two distinct variants due to base-pair mismatching between the two species causing a Tm shift of 2 °C. For testing the accuracy of this assay, a blind experiment was performed using a range of samples including some of the misidentified sample. The results showed a clear discrimination of the two variants. The results confirmed the re-classification of those samples into the correct genus, supporting the results with the specific primers.

All together these results proved the specificity and reliability of both techniques in the identification of *Ophiopogon* and *Liriope* samples. The PCR assays are limited by the requirement to design-specific primers for each known target plant and likely adulterant. The HRM assay has the ability to detect unknown contaminants provided they share the same genetic sequences and could be used to analyse admixtures in a single tube. However, genus-specific PCR primers and HRM are both powerful assays for a rapid genus-level screen without having to go through the entire DNA barcoding process for the identification of *Ophiopogon* and *Liriope* species. These two assays could be good tools for the discrimination of genus, species, or cultivars based on individual SNPs.

## 4. Materials and Methods

### 4.1. Plant Material and Total DNA Extraction

Fresh leaves were collected from 75 different species of *Ophiopogon* and *Liriope* at Brooksby Melton College (Melton Mowbray, Leicestershire, UK) from the UK National Plant Collections for *Ophiopogon* and *Liriope*. Details of the genus, species, and accession number are in Table S1. Samples were stored at −80 °C. DNA was extracted from 100 mg of frozen material, previously ground to a fine powder in liquid nitrogen with mortar and pestle, using DNeasy Plant Mini Kit (Qiagen Inc., Germantown, MD, USA) following the manufacturers’ guidelines.

### 4.2. PCR Protocols

PCRs were carried out using different primers as detailed in Table 2.

PCR reaction mixes contained 1X MyTaq Red Mix (Bioline), 0.2 μM of each forward and reverse primer, and 1 μL of gDNA as template. A G-Storm GS1 Thermal Cycler (G-Storm Ltd., Somerton, UK) was used with the following program:

- *rbcLa* PCR: initial denaturation step of 5 min at 95 °C followed by 35 cycles consisting of 30 s at 95 °C, 20 s at 52 °C, and 50 s at 72 °C, with a final extension period of 5 min at 72 °C.
- *Ophiopogon*-specific *rbcL* PCR: initial denaturation step of 5 min at 95 °C followed by 40 cycles consisting of 30 s at 95 °C, 30 s at 62.5 °C, and 45 s at 72 °C, with a final extension period of 5 min at 72 °C.
- *Liriope*-specific *rbcL* PCR: initial denaturation step of 5 min at 95 °C followed by 35 cycles consisting of 30 s at 95 °C, 30 s at 61 °C, and 30 s at 72 °C, with a final extension period of 5 min at 72 °C.
- HRM (generic) *rbcL* PCR: initial denaturation step of 5 min at 95 °C followed by 35 cycles consisting of 30 s at 95 °C, 30 s at 60 °C, and 30 s at 72 °C, with a final extension period of 5 min at 72 °C.

PCR products were run on 2% (*w*/*v*) agarose, 1X TBE gels with 1 μL SYBR^®^ Safe DNA Gel Stain (Invitrogen, Paisley, UK) at 100 V for 30 min and analysed in a Gel Doc™ EZ Gel Documentation System (BioRad, Oxford, UK).

### 4.3. DNA Sequence Analysis

Published *Liriope* and *Ophiopogon* rbcL DNA sequences were obtained from the National Center for Biotechnology Information (NCBI) GenBank database (http://www.ncbi.nlm.nih.gov/). A multi alignment was generated using CLC Main Workbench 7.5.1 software (Qiagen, Germantown, MD, USA).

### 4.4. Real-Time PCR Analysis

Each real-time PCR reaction contained 1 μL of gDNA, 1X Sensifast SYBR green Hi-Rox mix (Bioline), 0.1 μM of each forward and reverse primer in a total volume of 10 mL made up with sterile distilled water. A StepOnePlus™ Real-Time PCR thermocycler machine (Applied Biosystem) was used. Amplification conditions were as follows: 95 °C for 2 min followed by 40 cycles of 5 s at 95 °C and 30 s at the primer specific Ta (Table 1). The melting curve was obtained by melting the amplified template from 65 to 95 °C increasing the temperature by 0.5 °C per cycle. No-template controls were included. Three technical replicates were used for each sample. Internal StepOne software (Applied Biosystems) was used for the analysis of the results [24].

### 4.5. High-Resolution Melt Curve Analysis (HRM) Methods

HRM primers were designed to match the conserved sequences of the *rbcL* gene on either side of the genus-specific SNP (Figure 7). Each HRM real-time PCR reaction contained 1 μL of gDNA, 1X MeltDoctor™ HRM Master Mix (Applied Biosystem), 0.1 μM of each HRM_rbcL_forward and reverse primer (Table 1) in a total volume of 10 μL made up with sterile distilled water. A StepOnePlus™ Real-Time PCR thermocycler machine (Applied Biosystem) was used. Amplification conditions were as follows: 95 °C for 10 min followed by 40 cycles of 15 s at 95 °C and 30 s at 60 °C. The fluorescent data for PCR amplification was recorded during the extension step. The final melting curve was obtained by melting the amplified template 65 to 95 °C increasing the temperature by 0.3 °C per cycle with a 15 s hold time for each acquisition step. No-template controls were included. Three technical replicates were used for each sample.

HRM software (Applied Biosystem) has been used to analyse the results. For each sample, a melting curve plot, a melting peak plot and difference plot was generated [25].

## Figures and Tables

**Table 1:** Results of real-time PCR assay using *Ophiopogon*- and *Liriope*-specific primers. The *C*_t_ value obtained for each specific primer pair was normalised by subtracting the *C*_t_ value obtained for the same sample with the generic (HRM) primers, giving the Δ*C*_t_ (genus-specific − generic) value. The Δ*C*_t_ (genus-specific − generic) value for a reference standard was then subtracted from the Δ*C*_t_ genus-specific value for each sample, giving the ΔΔ*C*_t_ [(genus-specific − generic)_sample_ − (genus-specific − generic)_standard_] value. The reference standard for *Liriope* was chosen as Sample 624 and for *Ophiopogon* was Sample 678.

Sample Number	Δ*C*_t_ *Liriope*-Specific	Δ*C*_t_ *Ophiopogon*-Specific	ΔΔ*C*_t_ *Liriope*-Specific	ΔΔ*C*_t_ *Ophiopogon*-Specific
624	12.41	23.53	0	8.61
626	13.28	24.21	0.87	9.29
628	25.51	13.00	13.10	−1.92
633	12.28	23.64	−0.13	8.72
634	11.67	22.91	−0.74	7.99
667	24.74	15.17	12.33	0.25
677	24.46	13.87	12.05	−1.05
678	24.34	14.92	11.92	0
679	25.89	15.45	13.48	0.53
693	13.76	25.68	1.35	10.76
695	14.43	25.62	2.02	10.70
709	13.03	24.33	0.62	9.41
711	12.56	22.55	0.15	7.63

**Table 2:** List of primers with relative Ta and predict band size.

Primers	Sequences	Annealing Temperature (Conventional PCR)	Annealing Temperature (Real-Time PCR)	Amplicon Size (bp)
rbcLFw	ATGTCACCACAAACAGAGACTAAAGC	52 °C	N/A	700
rbcLRv	GTAAAATCAAGTCCACCRCG			
rbcL_Liriope_Fw	ATCCAAGTTGAAAGAGATAAATTA	61 °C	61 °C	180
rbcL_Liriope_Rv	AACGCATAAAAGGTTGTGAGTTA			
rbcL_Ophiopogon_Fw	ATCCAAGTTGAAAGAGATAAATTG	62.5 °C	64.5 °C	180
rbcL_Ophiopogon_Rv	AACGCATAAAAGGTTGTGAGTTC			
HRM_rbcL_Fw	CGCCTCATGGCATCCAAGT	61 °C	61 °C	80
HRM_rbcL_Rv	AATAGGGGACGACCATACTTG			

## Supplementary Materials

The following are available online at www.mdpi.com/2223-7747/6/4/53/s1. Figure S1: Multiple alignment of the *rbcLa* region of all of the *Liriope* and *Ophiopogon* samples tested, Figure S2: Agarose gel electrophoresis of PCR products of a re-sampled specimens using *Ophiopogon*- and *Liriope*-specific primers, Figure S3: Agarose gel electrophoresis of PCR products using HRM primers.

## Abbreviations

nrITS	nuclear ribosomal internal transcribed spacer
rbcL	large subunit of ribulose-1,5-bisphosphate carboxylase/oxygenase gene
HRM	high-resolution melt curve assay
