# Supplementary material for: Genus-Specific Real-Time PCR and HRM Assays to Distinguish Liriope from Ophiopogon Samples
Source: Plants (Basel). 2017 Oct 26;6(4):53. doi: 10.3390/plants6040053 (PMC5750629; doi:10.3390/plants6040053)
Supplement: Supplementary file 1 [file plants-06-00053-s001.zip › plants-231071-supplementary/plants-231071-supplementary-english-layout.pdf]

plants MDPI

Table S1. Samples taken from accessions in the UK National Collections of *Liriope* and *Ophiopogon*.

Sample Number	Accession number Brooksby Melton College	Genus	Species	Cultivar/hybrid	SNP
624	1996-54	*Liriope*	*exiliflora*		A
625	1996-02	*Liriope*	*graminifolia*	Alba	A
626	1996-03	*Liriope*	*graminifolia*	Intermedia	A
627	1996-05	*Liriope*	*graminifolia*	Koreana	A
628	1996-04	*Liriope*	*graminifolia*	Pre Alba	G
630	1996-01	*Liriope*	*graminifolia*		A
631	1996-46	*Liriope*	*minor*		A
632	1996-47	*Liriope*	*Or Ophiopogon herifu*		A
633	1997-43	*Liriope*	*muscari*	1103	A
634	1998-25	*Liriope*	*muscari*	'Monroe White'	A
636	1998-31	*Liriope*	*muscari*	'Silver Ribband'	A
637	1996-07	*Liriope*	*muscari*	Alba	A
638	1996-08	*Liriope*	*muscari*	Big Blue Riegal	A
639	1996-09	*Liriope*	*muscari*	Big Blue Tidwell	A
640	1996-10	*Liriope*	*muscari*	Blue Spire	A
641	1996-40	*Liriope*	*muscari*	BSWJ 561	A
642	1997-11	*Liriope*	*muscari*	Crested White	A
643	1996-12	*Liriope*	*muscari*	Curly Twist	A
644	1996-13	*Liriope*	*muscari*	Densiflora	A
645	1996-14	*Liriope*	*muscari*	Exaltation	A
646	1996-15	*Liriope*	*muscari*	Franklin Mint	A
647	1996-16	*Liriope*	*muscari*	Gigantea	A
648	1996-17	*Liriope*	*muscari*	Gilner White	A
649	1996-18	*Liriope*	*muscari*	Gold Banded	A
651	1996-42	*Liriope*	*muscari*	Grandiflora	A
652	1996-19	*Liriope*	*muscari*	Green Midget	A
653	1996-20	*Liriope*	*muscari*	Hawk Feathers	A
654	1996-21	*Liriope*	*muscari*	Ingwersens variety.	A
655	1996-22	*Liriope*	*muscari*	John Burch	A
656	1996-23	*Liriope*	*muscari*	Majestic	A
657	1996-26	*Liriope*	*muscari*	New Wonder	A
660	1996-28	*Liriope*	*muscari*	Purple Bouquet	A
661	1996-52	*Liriope*	*muscari*	Rocket	A
662	1996-49	*Liriope*	*muscari*	Royal Purple	A
663	1997-29	*Liriope*	*muscari*	Samantha	A
664	1997-30	*Liriope*	*muscari*	Sheffield	A
665	1996-50	*Liriope*	*muscari*	Silver Banded/Silver Dragon	A
666	1998-32	*Liriope*	*muscari*	Silver variegated	A
667	1997-37	*Liriope*	*muscari*	Silvery Midget	G
668	1996-33	*Liriope*	*muscari*	Sun Proof	A
669	1998-34	*Liriope*	*muscari*	Superba	A
670	1996-38	*Liriope*	*muscari*	Variegata alba	A
673	1996-41	*Liriope*	*muscari*	Wonder Evergreen	A
674	1996-35	*Liriope*	*muscari*	Xmas Tree	A
675	1996-06	*Liriope*	*muscari*		A
676	1997-45	*Liriope*	*spicata*	Alba	A
677	1998-44	*Liriope*	*spicata*		G
678	1996-01	*Ophiopogon*	*bodinieri*		G
679	1996-02	*Ophiopogon*	*chingii*		G
680	1996-03	*Ophiopogon*	*clarkei*		G

682	1996-05	*Ophiopogon*	*intermedius*	Shiroshiga Ryu	G
683	1997-29	*Ophiopogon*	*intermedius*	variegatus	G
684	1996-04	*Ophiopogon*	*Intermedius*		G
685	1997 -07	*Ophiopogon*	*jaburan*	Variegatus	G
686	1997-27	*Ophiopogon*	*jaburan*		G
688	1997-09	*Ophiopogon*	*japonicus*	Compactus	G
690	1996-12	*Ophiopogon*	*japonicus*	Little Tabby	G
691	1997-10	*Ophiopogon*	*japonicus*	Nippon	G
692	1997-11	*Ophiopogon*	*japonicus*	Tama ryu number 2	G
693	1998-31	*Ophiopogon*	*japonicus*	Torafu	A
695	1996-08	*Ophiopogon*	*japonicus*		A
697	1998-33	*Ophiopogon*	*planiscapus*	‘Black Dragon’	G
698	1998-32	*Ophiopogon*	*planiscapus*	‘Silver Edge’	G
699	2001-34	*Ophiopogon*	*planiscapus*	Black beard	G
700	1996-16	*Ophiopogon*	*planiscapus*	Leucanthus	G
701	1996-15	*Ophiopogon*	*planiscapus*	Nigrescens	G
702	1996-14	*Ophiopogon*	*planiscapus*		G
703	1996-17	*Ophiopogon*	*Sp. DF 86128*		G
704	1996-19	*Ophiopogon*	*wallichianus*		G
705	1997-25	*Ophiopogon*		‘Buncho’	G
706	1997-20	*Ophiopogon*		‘Fuko-ho-ryu’	G
708	1997-21	*Ophiopogon*		‘Rhyuko’	G
709	1997-22	*Ophiopogon*		‘Spring Gold’	A
711	1996-18	*Ophiopogon*		Stephen Tappler	A

Figure S1 supplied in pdf format

Figure S1. Multiple alignment of the *rbcLa* region of all of the *Lirope* and *Ophiopogon* samples tested. The first group of sequences represents reference sequences from the GenBank database.

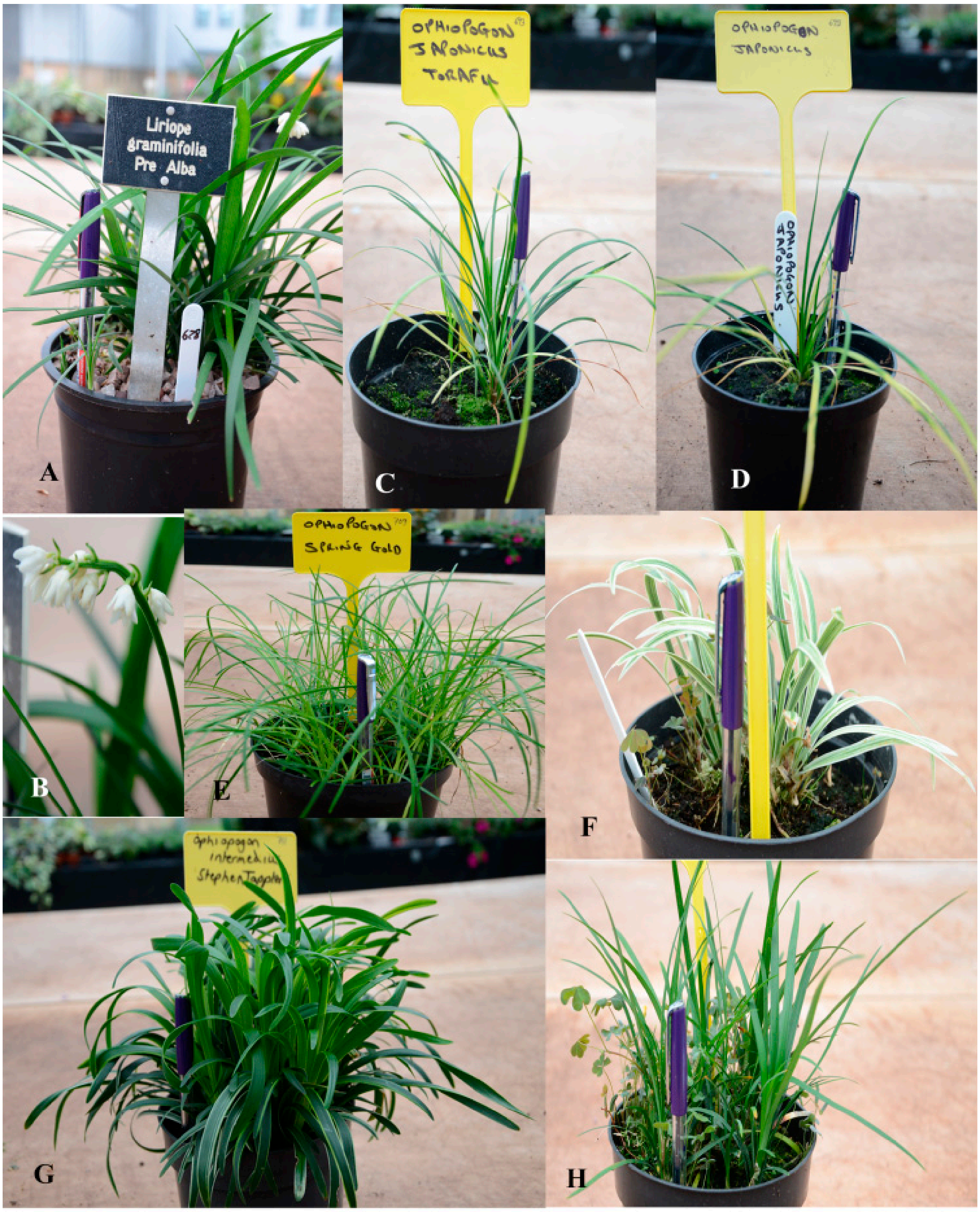

Figure S2. Photographs of misidentified specimens attributed to the incorrect genus. The original incorrect names are listed here. (**A**,**B**) Sample 628: *Liriope graminifolia*; (**C**) Sample 693: *Ophiopogon japonicus*; (**D**) Sample 695: *Ophiopogon japonicus*; (**E**) Sample 709: *Ophiopogon japonicus*.; (**F**) Sample 667: *Liriope muscari*; (**G**) Sample 711: *Ophiopogon intermedius*; (**H**) Sample 677: *Liriope spicata*.

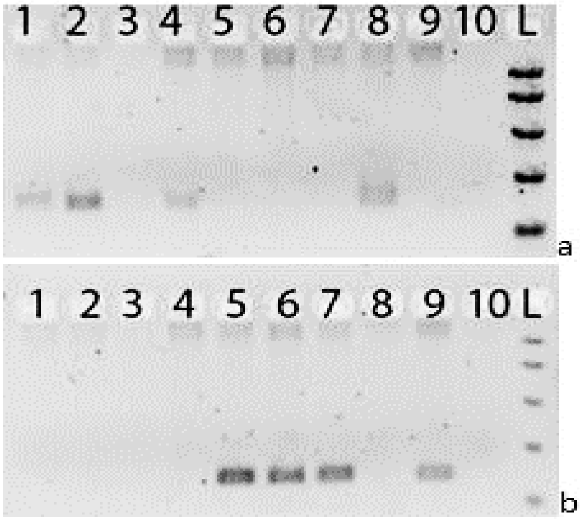

Figure S3. Agarose gel electrophoresis of PCR products of a re-sampled specimens using *Ophiopogon*- and *Liriope*-specific primers. (**a**) Testing the new collection of samples with *Liriope*-specific primers. (**b**) Testing the new collection of samples with Ophiopogon-specific primers. Gel lanes: 1. *Ophiopogon* Sample 693; 2. *Ophiopogon* Sample 695; 3. *Ophiopogon* Sample 709; 4. *Ophiopogon* Sample 711; 5. *Liriope* Sample 628; 6. *Liriope* Sample 667; 7. *Liriope* Sample 677; 8. *Liriope* Sample 625; 9. *Ophiopogon* Sample 692; 10. Negative (no template) control; L. Easy Ladder I (Bioline).

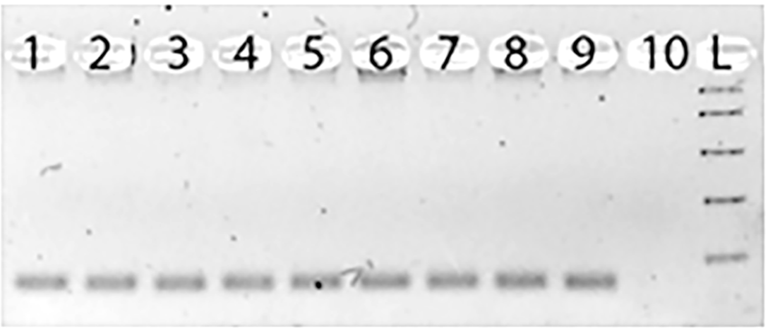

Figure S4. Agarose gel electrophoresis of PCR products using HRM primers. Gel lanes: 1. *Ophiopogon* Sample 678; 2. *Ophiopogon* Sample 679; 3. *Ophiopogon* Sample 693; 4. *Ophiopogon* Sample 695; 5. *Ophiopogon* Sample 709; 6. *Ophiopogon* Sample 711; 7. *Liriope* Sample 624; 8. *Liriope* Sample 628; 9. *Liriope* Sample 677; 10. Negative (no template) control; L. Easy Ladder I (Bioline).

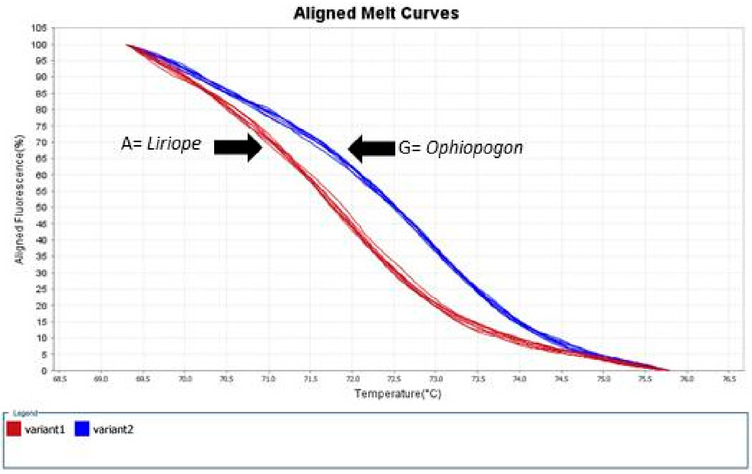

Figure S5. HRM assay. The melting curve plot shows two distinct variants. Variant 1: *Liriope* samples; Variant 2: *Ophiopogon* samples.
