# Supplementary figures and images for: Genus-Specific Real-Time PCR and HRM Assays to Distinguish Liriope from Ophiopogon Samples
Source: Plants (Basel). 2017 Oct 26;6(4):53. doi: 10.3390/plants6040053 (PMC5750629; doi:10.3390/plants6040053)

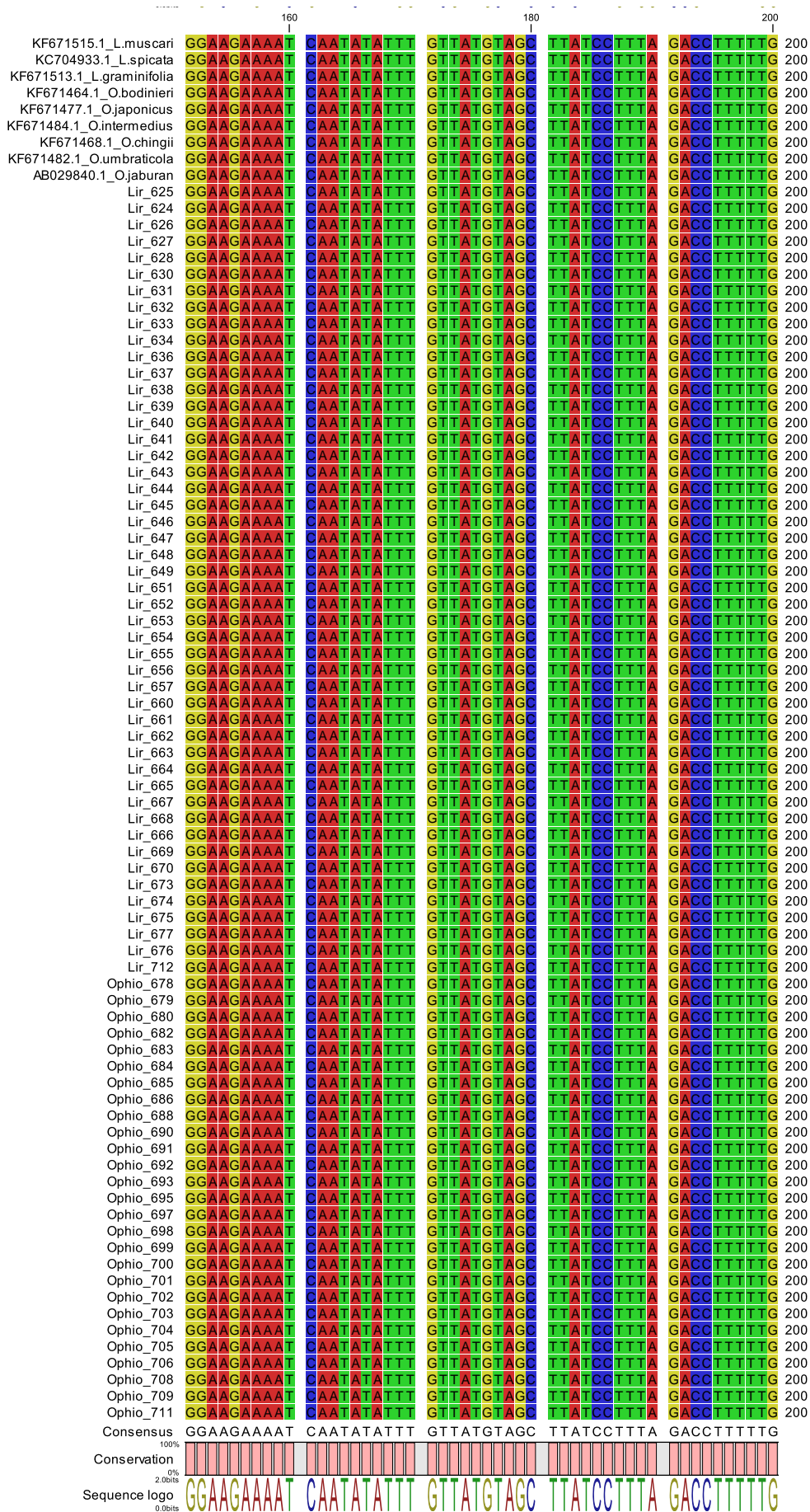



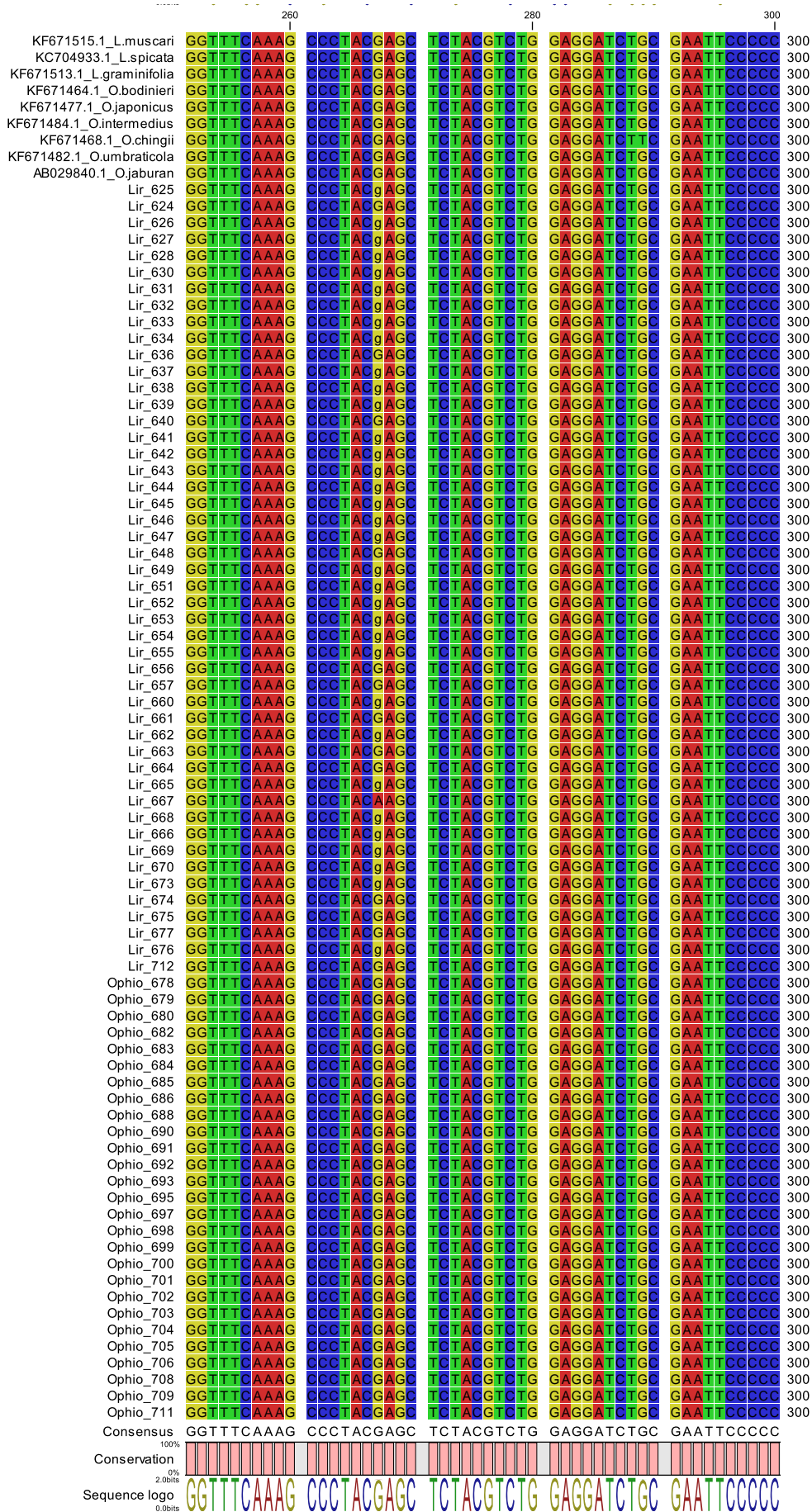

Supplement: Supplementary file 1 [file plants-06-00053-s001.zip › plants-231071-supplementary/plants-231071-supplementary-FigS1.pdf]
